# Crystallization and preliminary X-ray crystallographic analysis of polyphenol oxidase from *Juglans regia* (*jr*PPO1)

**DOI:** 10.1107/S2053230X1400884X

**Published:** 2014-05-28

**Authors:** Florime Zekiri, Aleksandar Bijelic, Christian Molitor, Annette Rompel

**Affiliations:** a Institut für Biophysikalische Chemie, Universität Wien, Althanstrasse 14, 1090 Wien, Austria

**Keywords:** tyrosinase, type 3 copper enzyme, *Juglans regia*, polyphenol oxidase

## Abstract

Tyrosinase is a type 3 copper enzyme that catalyzes the *ortho*-hydroxylation of monophenols to diphenols as well as their subsequent oxidation to quinones, which are precursors for the biosynthesis of melanins. The first plant tyrosinase from walnut leaves (*Juglans regia*) was purified to homogeneity and crystallized. During the purification, two forms of the enzyme differing only in their C-termini [*jr*PPO1(Asp^101^–Pro^444^) and *jrPPO1*(Asp^101^–Arg^445^)] were obtained. The most abundant form *jr*PPO1(Asp^101^–Arg^445^), as described in Zekiri *et al.* [*Phytochemistry* (2014 ▶), **101**, 5–15], was crystallized, resulting in crystals that belonged to space group *C*121, with unit-cell parameters *a* = 115.56, *b* = 91.90, *c* = 86.87 Å, α = 90, β = 130.186, γ = 90°, and diffracted to 2.39 Å resolution. Crystals were only obtained from solutions containing at least 30% polyethylene glycol 5000 monomethyl ether in a close-to-neutral pH range.

## Abstract 2

The crystallization and preliminary X-ray crystallographic analysis of a plant PPO exhibiting monophenolase activity from *J. regia* (*jr*PPO1) in its active form (Asp^101^–Arg^445^) are reported.

## 1. Introduction   

Tyrosinases (EC 1.14.18.1 and 1.10.3.1) are type 3 copper enzymes that are widely distributed in nature, occurring in many organisms including plants, mammals, fungi and bacteria [for reviews on polyphenol oxidases (PPOs) including tyrosinases, see van Gelder *et al.*, 1997 ▶; Marusek *et al.*, 2006 ▶; Mayer, 2006 ▶]. They are bifunctional enzymes which use molecular oxygen to catalyze the oxidation of various monophenols to *o*-diphenols (cresolase/monophenolase activity; EC 1.14.18.1) and the subsequent oxidation of *o*-diphenols to the corresponding *o*-quinones (catecholase/diphenolase activity; EC 1.10.3.1), which are precursors for the biosynthesis of melanins. Tyrosinases belong to a larger family of enzymes named polyphenol oxidases, which also include catechol oxidases and laccases. Catechol oxidases are type 3 copper enzymes that exclusively catalyze the oxidation of *o*-diphenols to *o*-quinones (catecholase/diphenolase activity; EC 1.10.3.1) but lack cresolase/monophenolase activity (EC 1.14.18.1). Laccases (EC 1.10.3.2) are multi-copper enzymes that oxidize a wide range of compounds including aminophenols, monophenols, *o*-diphenols and *p*-diphenols by removing single electrons from the reducing group of the substrate and generating free radicals (Mayer & Harel, 1979 ▶; Sanchez-Amat & Solano, 1997 ▶).

To date, only crystal structures of bacterial and fungal tyrosinases have been reported in the literature. The first crystal structure of a tyrosinase was obtained from the bacterium *Streptomyces castaneoglobisporus* and was published in 2006 (Matoba *et al.*, 2006 ▶), followed by the crystal structure of the bacterial tyrosinase from *Bacillus megaterium* (Sendovski *et al.*, 2011 ▶). Two crystal structures of fungal tyrosinase from the mushroom *Agaricus bisporus*, *ab*PPO3 (UniProt C7FF04) and *ab*PPO4 (UniProt C7FF05) (Ismaya *et al.*, 2011 ▶; Mauracher, Molitor, Al-Oweini *et al.*, 2014 ▶), have recently been published. To date, only two crystal structures of catechol oxidases have been reported: the 39 kDa isoform of the *Ipomoea batatas* (sweet potato) catechol oxidase (UniProt Q9ZP19; Klabunde *et al.*, 1998 ▶) and the catechol oxidase from the grape *Vitis vinifera* (UniProt P4331; Virador *et al.*, 2010 ▶).

The active site of tyrosinase consists of two copper ions, each coordinated by three conserved histidine residues. It has been proposed that tyrosinases are involved in defence mechanisms against pathogens because of the bacteriostatic properties of *o*-quinones and melanins. The study of tyrosinases in plants has focused on their role in the process of post-harvest browning, whereby cut or damaged plant tissues turn brown owing to the polymerization of tyrosinase-derived quinones (Queiroz *et al.*, 2008 ▶). Walnut presents an interesting model to further explore the function of tyrosinase in plants based on the high content of various phenolic compounds in walnut tissues (Colaric *et al.*, 2005 ▶; Solar *et al.*, 2006 ▶; Araji *et al.*, 2014 ▶).

Recently, a tyrosinase from walnut leaves (*Juglans regia*; *jr*PPO1; UniProt COLU17) was isolated and characterized as having a monophenolase activity (Escobar *et al.*, 2008 ▶; Araji *et al.*, 2014 ▶; Zekiri *et al.*, 2014 ▶), thus making *jr*PPO1 an attractive enzyme for crystallographic studies of a plant tyrosinase. Although it exhibits monophenolase activity, *jr*PPO1 has the highest sequence identity to catechol oxidase from *V. vinifera* (sequence identity 62%; UniProt P4331; Virador *et al.*, 2010 ▶) and has only weak identity (<20%) to bacterial and fungal tyrosinases (Matoba *et al.*, 2006 ▶; Ismaya *et al.*, 2011 ▶; Sendovski *et al.*, 2011 ▶; Mauracher, Molitor, Michael *et al.*, 2014 ▶). Here, the crystallization and preliminary crystallographic analysis of *jr*PPO1 are reported. The study is performed to investigate possible structural differences between plant tyrosinases and plant catechol oxidases.

## 2. Materials and methods   

### 2.1. Sample preparation   

The isolation and purification process of the enzyme has been described in detail by Zekiri *et al.* (2014 ▶). Briefly, extraction of the enzyme from the natural source (walnut leaves) was performed using the method developed by Mauracher, Molitor, Michael *et al.* (2014 ▶) based on detergent and soluble polymer polyethylene glycol (PEG) phase separations. The active tyrosinase was purified to homogeneity by fast protein liquid chromatography (FPLC) using several ion-exchange columns. During the purification, two forms of the enzyme differing only in their C-termini [*jr*PPO1(Asp^101^–Pro^444^) and *jr*PPO1(Asp^101^–Arg^445^)] were obtained and were determined by peptide sequencing applying nanoUHPLC-ESI-MS/MS. The purity of *jr*PPO1 was monitored by SDS–PAGE. For crystallization experiments *jr*PPO1(Asp^101^–Arg^445^), the most abundant form, was concentrated to 10 mg ml^−1^ in 20 m*M* HEPES pH 7.5.

### 2.2. Protein crystallization   

**Figure 1:**
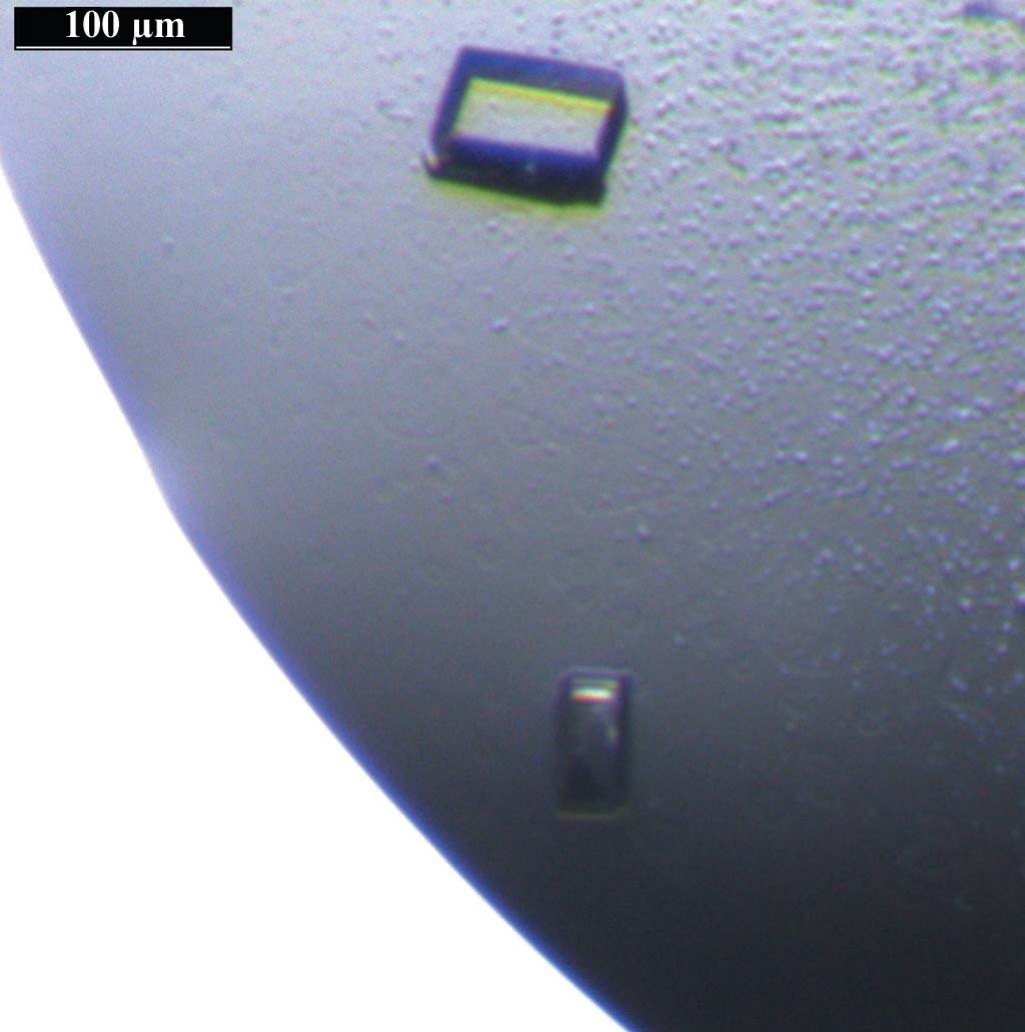
Plate-shaped crystals obtained using 30% PEG 5000 MME, 200 m*M* ammonium sulfate, 100 m*M* MES pH 6.5.

Initial screening for crystallization was carried out by the sitting-drop vapour-diffusion method (96-well CrystalQuick plates, Greiner Bio-One) employing a nanodispenser robot (Gryphon, Art Robbins). Initial hits were obtained by screening over a broad variety of commercially available screening kits (JBScreen Classic 1–10 and JBScreen Membrane 1–3 from Jena Bioscience) mixing the protein solution with the reservoir solution in a 1:2 ratio. Further optimization of the crystallization conditions was performed manually in 15-well EasyXtal plates (Qiagen) applying the hanging-drop vapour-diffusion method at 293 K. Single crystals suitable for diffraction measurements were obtained after 2–3 d by mixing 1 µl protein solution (10 mg ml^−1^) with 0.5 µl reservoir solution and equilibrating against 500 µl reservoir solution consisting of 30% PEG 5000 MME (MME, monomethyl ether), 200 m*M* ammonium sulfate, 100 m*M* MES pH 6.5 (Fig. 1 ▶). The crystals stopped growing after approximately 6 d.

### 2.3. Data collection and processing   

Single crystals were harvested by transferring them with a cryoloop (10 µm, 0.1 × 0.2 mm; Hampton Research) into a 0.5 µl drop of cryoprotectant solution (30% PEG 5000 MME, 20% glycerol, 200 m*M* ammonium sulfate, 100 m*M* MES pH 6.5) before flash-cooling them in liquid nitrogen. X-ray diffraction measurements of about 20 crystals of suitable size were carried out at DESY (Hamburg, Germany) on the monochromatic (1.033 Å) beamline P11. Diffraction data were collected with a PILATUS 6M detector at 100 K with an oscillation range of 0.2° and an exposure time of 0.190 s. The best crystal diffracted to 2.39 Å resolution using a crystal-to-detector distance of 435 mm. Data processing was carried out using the *XDS* program package (Kabsch, 2010 ▶). The space group was determined using the program *POINTLESS* from the *CCP*4 suite (v.6.4.0; Winn *et al.*, 2011 ▶). The data set presented here had strong anisotropy as indicated by *phenix.xtriage* from the *PHENIX* suite (v.1.8.4; Adams *et al.*, 2010 ▶) and was therefore truncated (discarding reflections falling outside a specific ‘ellipsoid’ with dimensions 1/2.4, 1/2.4 and 1/2.4 Å along a*, b* and c*, respectively) and anisotropically scaled using the *Diffraction Anisotropy Server* at UCLA MBI (Strong *et al.*, 2006 ▶).

## 3. Results and discussion   

By applying the method described by Zekiri *et al.* (2014 ▶), the enzyme was purified to homogeneity. Initial attempts to crystallize the most abundant form *jrPPO1*(Asp^101^–Arg^445^), as described in Zekiri *et al.* (2014 ▶), covering a wide range of crystallization conditions proved to be very successful. Crystals suitable for X-ray diffraction experiments were obtained using 30% PEG 5000 MME, 200 m*M* ammonium sulfate, 100 m*M* MES pH 6.5 (Fig. 1 ▶). The crystals were plate shaped and of reasonable size (100 × 50 × 10 µm) for X-ray diffraction analysis. Crystallization of *jr*PPO1 has been achieved by conditions that differ in almost all crystallization parameters (pH, precipitation agent, temperature or additives) from those reported previously for PPO crystallization. The utilization of PEG as a precipitation agent is the only constant. All of the published crystallization conditions of fungal (Ismaya *et al.*, 2011 ▶; Mauracher, Molitor, Al-Oweini *et al.*, 2014 ▶) and bacterial tyrosinases (Matoba *et al.*, 2006 ▶; Sendovski *et al.*, 2011 ▶) as well as plant catechol oxidases (Klabunde *et al.*, 1998 ▶; Virador *et al.*, 2010 ▶) contain PEG as precipitation agent with different molecular masses (PEG 4000–8000).

Processing statistics for the X-ray diffraction measurements are presented in Table 1 ▶. The crystals belonged to space group *C*121, with unit-cell parameters *a* = 115.56, *b* = 91.90, *c* = 86.87 Å, α = 90, β = 130.186, γ = 90°, and diffracted to a maximum resolution of 2.39 Å. The solvent content was determined with the Matthews formula using a molecular mass of 39.047 kDa (Zekiri *et al.*, 2014 ▶). This gives a Matthews coefficient (Matthews, 1968 ▶) of 2.26 Å^3^ Da^−1^ and a solvent content of 45.52% assuming the presence of two monomers per asymmetric unit.

We are currently attempting to solve the crystal structure by using molecular replacement (MR). Two models for MR are available: *V. vinifera* catechol oxidase (sequence identity 62%; UniProt P4331; Virador *et al.*, 2010 ▶) and *I. batatas* catechol oxidase (sequence identity 56%; UniProt Q9ZP19; Klabunde *et al.*, 1998 ▶). The data set was anisotropic and was therefore truncated and anisotropically scaled. However, both the truncated and original data sets will be used in refinement to determine the best produced electron-density map, because *phenix.refine* carries out anisotropic scaling by default; thus, the truncated and pre-scaled data could be redundant, leading to a featureless map.

## Figures and Tables

**Table 1:** Data-collection and processing statistics for *jr*PPO1 crystals Values in parentheses are for the highest resolution shell.

Space group	*C*121
Wavelength (Å)	1.033
No. of images	900
Oscillation (°)	0.3
Resolution range (Å)	29.10–2.39 (2.48–2.39)
Completeness (%)	99.04 (96.68)
*R*_merge_ †	0.135 (0.514)
〈*I*/σ(*I*)〉	8.3 (2.7)
Multiplicity	5.04 (4.83)
Unit-cell parameters (Å, °)	*a* = 115.56, *b* = 91.90, *c* = 86.87, α = 90, β = 130.186, γ = 90
*R*_p.i.m._ ‡	0.067 (0.261)
CC_1/2_	0.994 (0.938)
No. of reflections collected	137555 (12784)
No. of unique reflections	27289 (2648)

† *R*_merge_ = 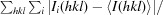 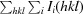.

‡ *R*_p.i.m._ = 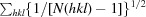 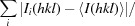 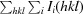, where *I_i_*(*hkl*) is the *i*th observation of reflection *hkl* and 〈*I*(*hkl*)〉 is the weighted average intensity for all observations of reflection *hkl*.
